# FishDB: an integrated functional genomics database for fishes

**DOI:** 10.1186/s12864-020-07159-9

**Published:** 2020-11-17

**Authors:** Liandong Yang, Zetan Xu, Honghui Zeng, Ning Sun, Baosheng Wu, Cheng Wang, Jing Bo, Lin Li, Yang Dong, Shunping He

**Affiliations:** 1 State Key Laboratory of Freshwater Ecology and Biotechnology, Institute of Hydrobiology, Chinese Academy of Sciences, Wuhan, 430072 China; 2 State Key Laboratory for Conservation and Utilization of Bio-Resources in Yunnan, Yunnan Agricultural University, Kunming, 650201 China; 3 University of Chinese Academy of Sciences, Beijing, 100049 China; 4 Institute of Deep-sea Science and Engineering, Chinese Academy of Sciences, Sanya, 572000 China; 5 Center for Excellence in Animal Evolution and Genetics, Chinese Academy of Sciences, Kunming, 650223 China

**Keywords:** Fish, Genome, Transcriptome, Evolution, Adaptation

## Abstract

**Background:**

Hundreds of genomes and transcriptomes of fish species have been sequenced in recent years. However, fish scholarship currently lacks a comprehensive, integrated, and up-to-date collection of fish genomic data.

**Results:**

Here we present FishDB, the first database for fish multi-level omics data, available online at http://fishdb.ihb.ac.cn. The database contains 233 fish genomes, 201 fish transcriptomes, 5841 fish mitochondrial genomes, 88 fish gene sets, 16,239 miRNAs of 65 fishes, 1,330,692 piRNAs and 4852 lncRNAs of *Danio rerio*, 59,040 Mb untranslated regions (UTR) of 230 fishes, and 31,918 Mb coding sequences (CDS) of 230 fishes. Among these, we newly generated a total of 11 fish genomes and 53 fish transcriptomes.

**Conclusions:**

This release contains over 410,721.67 Mb sequences and provides search functionality, a BLAST server, JBrowse, and PrimerServer modules.

**Supplementary Information:**

The online version contains supplementary material available at 10.1186/s12864-020-07159-9.

## Background

Fish are the largest group of vertebrates, covering over one-half of the world’s living vertebrates [1]. Considering the vast diversity of species and morphology, fish have received intense attention from scholars and the public, as they are important to both scientific research and aquaculture. The availability of fish genomes and transcriptomes will provide valuable resources for ichthyological research. However, fish scholarship currently lacks a comprehensive, integrated, up-to-date collection of fish omics data.

Currently, at least 222 fish genomes have been sequenced and deposited in public databases, including the NCBI genome database [2], Ensembl [3], UCSC [4], SalmoBase [5], GCGD [6], and cBARBEL [7]. Because the cost of whole-genome sequencing has decreased, many whole-genome sequencing projects on fish have been performed in recent years, usually by small research groups with technical support from private companies. Although the genomes are often required to be submitted to the NCBI genome database before publication, the database is not scheduled to be updated frequently in the future. More importantly, many more genomes have already been sequenced, but the necessary studies take time to publish, leaving many genomes unavailable to the research community. In this way, ichthyological research has been severely hampered for lack of a comprehensive, integrated, and up-to-date collection of fish omics database.

Here, we generated FishDB (http://fishdb.ihb.ac.cn), which is intended to meet the needs of the fish scholarship community. It is especially suitable for studies on taxonomy, phylogeny, evolution, development, and agriculture. As far as we know, FishDB gathers almost all of its fish genomes and most of its fish transcriptomes from public databases. We also included a total of 11 fish genomes and 53 fish transcriptomes collected by our group, which have not been accessible previously. FishDB provides not only widely used web-services such as a search tool, BLAST, JBrowse, and PrimerServer, but also a platform for comparative genomics analysis on orthologs.

## Construction and content

### Data sources

FishDB integrates fish gene data from as many as dozens of databases (Table 1). Here, we generated a total of 11 fish genomes and 53 fish transcriptomes for the first time.

**Table 1:** Summary of the data content of FishDB

Category	Species	Total Sequences (Mb)
Genome	233	258,892.84
Transcriptome	201	55,586.52
Mitogenomes	5841	137.62
EST	136	3889.03
Ortholog	48	1218.18
miRNA	65	0.99
piRNA	1	33.81
LncRNA	1	3.89
UTR	230	59,040.01
CDS	230	31,918.78

Most of the fish genomes were obtained from the genome database in NCBI [2], Ensembl [3], UCSC [4], EFish, SalmoBase [5], GCGD [6], and cBARBEL [7] (Supplementary Data S1). We also assembled 11 new genomes of comparable quality to those of the other fishes (Supplementary Data S2). All individual fish were euthanized, which was approved by the Institutional Animal Care and Use Committee of Institute of Hydrobiology, Chinese Academy of Sciences (Approval ID: Y21304501). Fish were purchased from a commercial aquarium (Wuhan Shengdajiahe Aquarium). One individual of each fish species was collected. Adult fishes were euthanized individually by immersion in water baths in a 5-L holding tank with aerated water containing 500 mg/L of MS-222 (Sigma). When the fish died, their muscle tissue was collected for sequencing. This generated a total of 233 fish genomes (Table 2). Among them, we obtained annotation files of genes for a total of 88 fish genomes (Supplementary Data S3) (Fig. 2a and b).

**Table 2:** The distribution of fish genome resource

Database	Species	Genome with Gene Sets	URL
NCBI (Genome)	294	74	https://www.ncbi.nlm.nih.gov/genome/
Ensembl	48	48	https://asia.ensembl.org/index.html
UCSC	10	10	https://genome.ucsc.edu/
EFish	3	2	https://efishgenomics.integrativebiology.msu.edu/
SalmoBase	2	2	https://salmobase.org/
GCGD	1	1	http://bioinfo.ihb.ac.cn/gcgd/php/index.php
cBARBEL	1	1	http://catfishgenome.org
New generated	11	11	–
FishDB	303	91	http://fishdb.ihb.ac.cn

Assembled fish transcriptomes were downloaded from the NCBI TSA (Transcriptome Shotgun Assemblies) database (Supplementary Data S4). We generated a total of 53 new transcriptomes sampled from tissues including muscle, brain, liver, kidney, and heart, which were then assembled using Trinity [8] with default parameters (Supplementary Data S5). We further collected a total of 49,406 raw RNA-seq from NCBI SRA (Sequence Read Archive) database (Supplementary Data S6).

Fish mitochondrial genomes were collected from MitoFish (Mitochondrial Genome Database of Fish) [9, 10]. A total of 2726 complete mtDNA sequences from 2726 fish species were obtained. We further downloaded a total of 8094 complete mtDNA sequences from 3121 fish species (Supplementary Data S7).

Expressed sequence tags (ESTs) of 136 fish were obtained from the EST database in NCBI [2].

Fish orthologs were downloaded from Ensembl (release 96; May 2019) using BioMart [11] and a total of 19,310 orthologs between zebrafish and at least one other fish species were downloaded (Supplementary Data S8) (Fig. 2c).

Fish miRNA sequences were collected from the miRBase [12], Ensembl (release 96; May 2019), and also obtained from the supplemental materials of published references when the miRNA sequences were not deposited into miRBase. In total, the miRNAs from 65 fish were stored in FishDB (Supplementary Data S9).

For piRNA and long noncoding RNA (lncRNA), a total of 1,330,692 piRNAs and 4852 lncRNAs of *Danio rerio* were downloaded from piRBase [13] and NONCODE [14], respectively.

Coding sequences (CDS) and untranslated regions (UTR).

We also obtained UTR sequences of five fish from the UTRBase [15]: *Danio rerio*, *Oncorhynchus mykiss*, *Oryzias latipes*, *Salmo salar*, and *Takifugu rubripes*. We also predicted CDS and UTR using TransDecoder from transcriptome sequences, producing CDS and UTR sequences for a total of 230 fish. In addition, we obtained CDS and UTR sequences from 48 fish genomes predicted by Ensembl. Collectively, CDS and UTR sequences from a total of 230 fish species were collected in FishDB (Supplementary Data S10).

## Utility and discussion

### Structure of FishDB

**Fig. 1:**
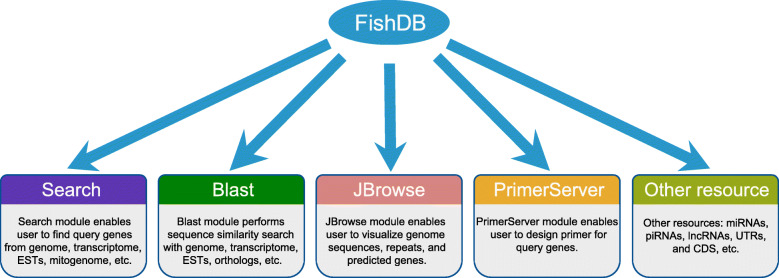
The structure of FishDB, which provides Search, Blast, JBrowse, PrimerServer, Ortholog, and fish gene information

FishDB offers web services including a search tool, BLAST, JBrowse, and PrimerServer. The gene information for noncoding RNA (ncRNA), microRNA (miRNA), UTRs, and CDSs was collected and stored in the FishDB database (Fig. 1).

### Search

The search module enables the user to collect interesting information, such as sequences, from genomes, transcriptomes, genes, ESTs, mitogenomes, and orthologos using either a gene name or geneID. In addition to sequence information, users could also find relevant information from the gene. The search results provide users with links to NCBI records.

### Blast

**Fig. 2:**
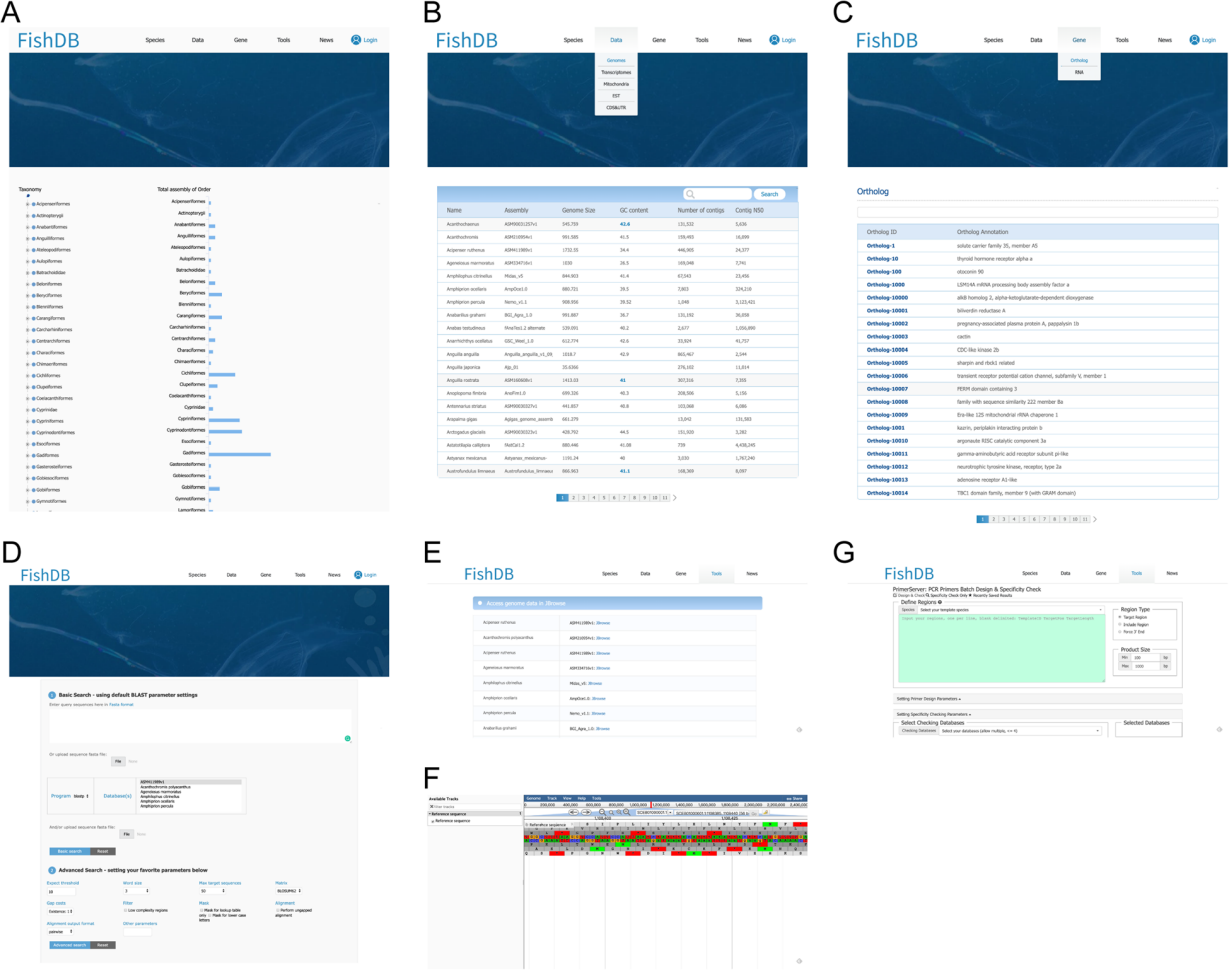
A overview of FishDB. **a** List of species number in each fish order. **b** Page of genomic data of fishes. **c** Page of ortholog in fish genomes. **d** The BLAST tool. **e** Page of JBrowse. **f** A example of JBrowse. **g** The primer server page

The blast module performs sequence similarity search employing a web-based BLAST server [16]. Users can use nucleotide BLAST (BLASTN and TBLASTN) searches against the 233 fish genomes, 201 fish transcriptomes, 136 fish ESTs, and 88 fish OGSs, and they can use amino acid BLAST (BLASTX and TBLASTX) searches against the 88 fish protein sequences (Fig. 2d).

### JBrowse

The JBrowse module enables users to visualize the 88 fish genomes [17], which is a related browser to the conventional CGI-based genome browser (GBrowse). This genome browser enables users to find and explore the 88 fish genome sequences and annotation information easily. Three main tracks, including CDS, mRNA, and exon, are integrated for all fish genomes. Users can find various tracks and search genomic features inside in the reference genome, including transposable elements,gene models, and repeats (Fig. 2e and f).

### PrimerServer

The PrimerServer module helps users design primers that are particular to polymerase chain reaction experiments (PCR). We used Primer3 [18] to produce candidate primer pairs for the sequences of given template. We also integrated Primer Blaster, a specific tool, to test the specificity of each primer pair. The designed primer sequences can be downloaded as fasta format. (Fig. 2g).

## Conclusions

We have built the Fish Genome Database (FishDB), which provides a central portal for genomics, transcriptomics, genetics, and evolutionary biology of fish. FishDB stores various sequences, including genomes, transcriptomes, mitochondrial genomes, ESTs, orthologs, noncoding RNAs, UTRs, and CDSs of fish species. The database also provides query, visualization, and primer design tools including BLAST, JBrowse, and PrimerServer. FishDB will be continuously updated when new genome, transcriptome, and genetic datasets of fish become available, and more enhanced functionality will be possible in the future to generate a more valuable resource for promoting comparative genomics, transcriptomes, and evolutionary biology studies.

## Supplementary Information

**Additional file 1 : Supplementary Data S1.** The fish genomes downloaded from public databases in FishDB.

**Additional file 2 : Supplementary Data S2.** The fish genomes newly generated from our lab in FishDB.

**Additional file 3 : Supplementary Data S3.** The fish gene sets in FishDB.

**Additional file 4 : Supplementary Data S4.** The fish transcriptomes downloaded from Transcriptome Shotgun Assembly (TSA) in FishDB.

**Additional file 5 : Supplementary Data S5.** The fish transcriptomes newly generated from our lab in FishDB.

**Additional file 6 : Supplementary Data S6.** The RNA-seq data sets of fish transcriptomes downloaded from Sequence Read Archive (SRA) in FishDB.

**Additional file 7 : Supplementary Data S7.** The fish mitochondrial genomes obtained from MitoFish and NCBI in FishDB.

**Additional file 8 : Supplementary Data S8.** Orthologs between zebrafish and at least one other fish species from Ensembl.

**Additional file 9 : Supplementary Data S9.** The fish miRNAs in FishDB.

**Additional file 10 : Supplementary Data S10.** The UTRs and CDSs of fishes in FishDB.

## Data Availability

FishDB can be accessed at http://fishdb.ihb.ac.cn. All data used in this study are available from Supplementary Data S1, S3, S4, S6, S7, and S8.

## Abbreviations

lncRNAs: Long non-coding RNAs
Mb: Millions of base pairs
UTR: Untranslated region
CDS: Coding sequences
TSA: Transcriptome shotgun assemblies
SRA: Sequence read archive
ESTs: Expressed sequence tags
